# AI Research Funding Portfolios and Extreme Growth

**DOI:** 10.3389/frma.2021.630124

**Published:** 2021-04-06

**Authors:** Ilya Rahkovsky, Autumn Toney, Kevin W. Boyack, Richard Klavans, Dewey A. Murdick

**Affiliations:** ^1^Center for Security and Emerging Technology (CSET), Georgetown University, Washington, DC, United States; ^2^SciTech Strategies, Inc., Albuquerque, NM, United States; ^3^SciTech Strategies, Inc., Wayne, PA, United States

**Keywords:** research analysis, research portfolio analysis, forecasting, artificial intelligence, machine learning, map of science, research funding agencies

## Abstract

Our work analyzes the artificial intelligence and machine learning (AI/ML) research portfolios of six large research funding organizations from the United States [National Institutes of Health (NIH) and National Science Foundation (NSF)]; Europe [European Commission (EC) and European Research Council (ERC)]; China [National Natural Science Foundation of China (NNSFC)]; and Japan [Japan Society for the Promotion of Science (JSPS)]. The data for this analysis is based on 127,000 research clusters (RCs) that are derived from 1.4 billion citation links between 104.8 million documents from four databases (Dimensions, Microsoft Academic Graph, Web of Science, and the Chinese National Knowledge Infrastructure). Of these RCs, 600 large clusters are associated with AI/ML topics, and 161 of these AI/ML RCs are expected to experience extreme growth between May 2020 and May 2023. Funding acknowledgments (in the corpus of the 104.9 million documents) are used to characterize the overall AI/ML research portfolios of each organization. NNSFC is the largest funder of AI/ML research and disproportionately funds computer vision. The EC, RC, and JSPS focus more efforts on natural language processing and robotics. The NSF and ERC are more focused on fundamental advancement of AI/ML rather than on applications. They are more likely to participate in the RCs that are expected to have extreme growth. NIH funds the largest relative share of general AI/ML research papers (meaning in areas other than computer vision, natural language processing, and robotics). We briefly describe how insights such as these could be applied to portfolio management decision-making.

## 1. Introduction

The research funding portfolios of large, government-sponsored organizations provide insight into the competitive research landscape and the corresponding research opportunities (or threats) that are being addressed in preparation for the future. In this study, we offer examples of these portfolios in order to highlight important details about each organization's funding portfolio.

Forecasting is a potentially helpful part of this process even if it does not guarantee success on its own (Gerstner, 1972). Forecasting is a process that leaders, experts, and analysts employ to estimate the probability of future events, relative states, or trends based on past and present data. Ideally, forecasts are data-driven and clearly described with a well-defined unit of analysis, time frame, occurrence probability or confidence interval, and, if possible, relevant conditional factors. Assumptions, opinions, and informal “gut instincts” from subject matter experts and non-experts alike can substitute for well-defined forecasts when they are absent, which may reduce the utility and accountability of these judgments (Tetlock, 2017).

The unit of analysis used in this study is called a research cluster (RC) and consists of a group of articles that have been linked by their citations. RCs are based on the principles laid out by Kuhn (1970). Kuhn referred to this unit of analysis as a “research community,” but he did not recommend that one use researchers to identify these communities. Rather, he suggested using their communications–specifically the claims they make in their articles that build upon separate claims made in other articles. The corpus used to create these RCs includes documents from Clarivate's Web of Science (WOS)^1^, Digital Science's Dimensions (DS), Microsoft Academic Graph (MAG), and the Chinese National Knowledge Infrastructure (CNKI). The data include ~1.4 billion citation links—instances where a researcher is communicating that they are using ideas, methods, results, or in any other way building on the work of other researchers. The large-scale clustering solution for creating RCs from these citation links builds upon the work of others (Waltman and Van Eck, 2012, 2013; Sjögårde and Ahlgren, 2018).

The method for forecasting the publication growth of each RC uses a recently developed forecasting methodology (Klavans et al., 2020). This forecasting method is transparent (i.e., the methods are described in the article) and was tested using data from the Scopus database (roughly 50 million papers) and separately using the merged dataset of WOS, DS, MAG, and CNKI. We applied this method to the RCs inferred from our merged database (roughly 104.9 million documents as of October 2020).

We also address other technical issues. The capabilities of a funding organization in a specific area of research may be over- or underestimated because we are relying on acknowledgments in the open literature. In this study, we address this problem by providing concrete examples so that the reader (who may be more familiar with a specific organization) can come to their own conclusions. We will look at the funding portfolios of a few of the largest granting organizations in the world—U.S. National Institutes of Health (NIH), U.S. National Science Foundation (NSF), European Commission (EC), European Research Council (ERC), National Natural Science Foundation of China (NNSFC), and Japan Society for the Promotion of Science (JSPS).

Previous work in several areas is relevant to the study reported here. Rather than providing an extensive review, we point to other papers that have substantially reviewed recent work in large-scale clustering of the research literature (Boyack and Klavans, 2019), identification of emerging topics (Small et al., 2014), forecasting of growth in research topics (Klavans et al., 2020), and use of funding acknowledgments (Liu et al., 2020).

The paper will proceed as follows: details are given of our methods and data, followed by an explanation of the resulting RC framework and results. The final section discusses findings, implementation principles, and a brief discussion of limitations.

## 2. Materials and Methods

Using a comprehensive corpus of scholarly literature published worldwide, we aim to infer RCs from these publications and forecast the clusters' growth. In this section, we describe (1) the dataset of scientific publications used in our analysis, (2) the method we use to infer RCs using direct citation links present in the publications, and (3) the method we use to predict growth in each cluster using the cluster forecasting model proposed by Klavans et al. (2020).

### 2.1. Scholarly Literature Dataset

In order to generate a comprehensive network of publications through direct citation links, we combine four large-scale scholarly literature data sources. The combined dataset merges three (MAG, Dimensions, and WOS) of the four commonly used large citation databases (Visser et al., 2020) with a large Chinese-language citation database, CNKI, and thus accounts for a relatively large fraction of the world's scholarly journal literature. All years from the MAG and DS databases are included, CNKI content includes 2005 onward, and WOS indices include content primarily from 2000 onward. Ultimately, the data include publications from a wide breadth of publishers, authors, institutions, and languages. In total, the data sources contain over 382.8 million documents^2^.

**Figure 1:**
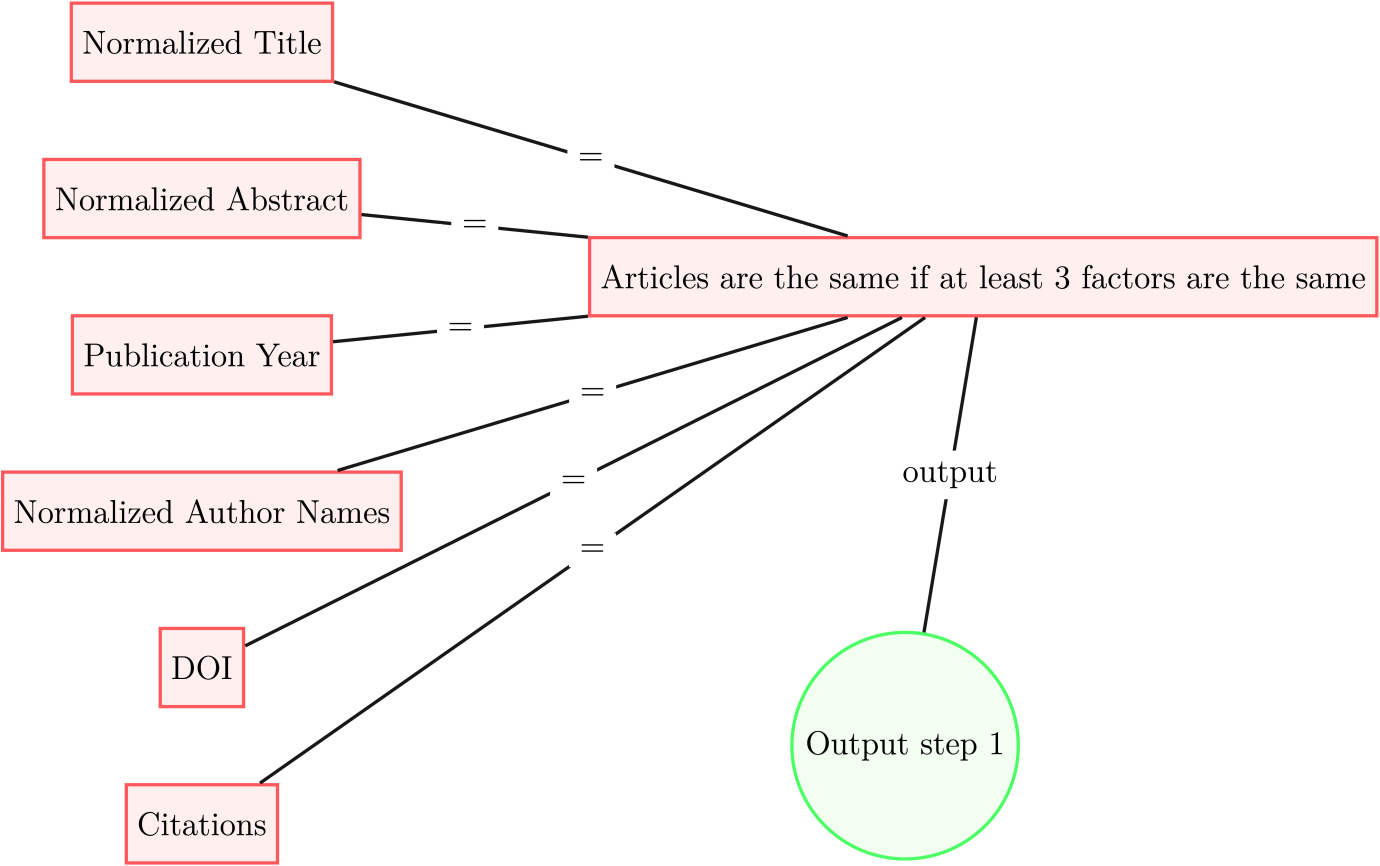
Article deduplication process scheme (step 1).

**Figure 2:**
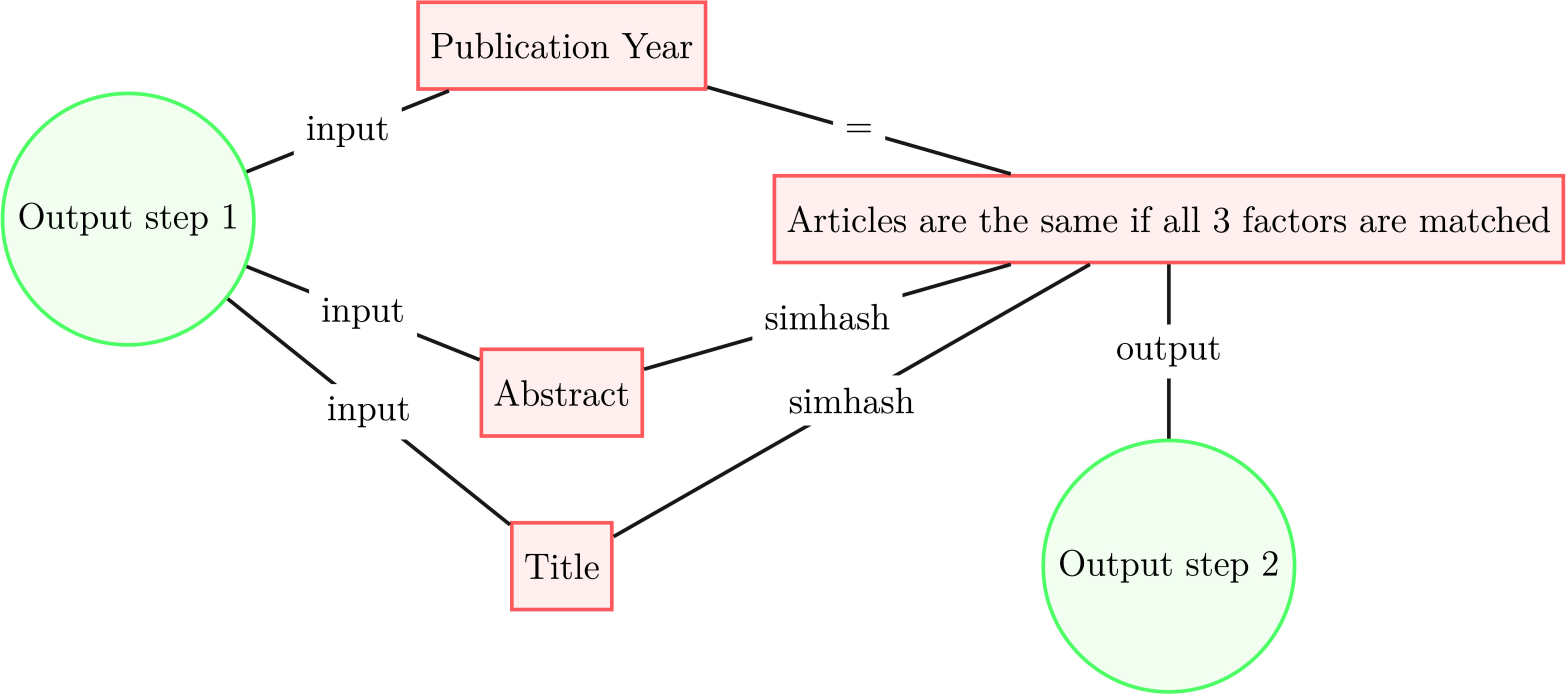
Article deduplication process scheme (step 2).

Since our datasets of scholarly literature do not share a publication identifier system, we deduplicate publications present in multiple data sources according to the following procedure presented in Figures 1, 2. In the first step, for each document in each data source, we select six identifiers: (1) normalized title, (2) normalized abstract, (3) publication year, (4) normalized surnames of authors, (5) DOI, and (6) citations. In steps 1, 2, and 4, the strings are normalized following the Normalization Form Compatibility Composition standard: unicode characters are decomposed by compatibility, then recomposed by canonical equivalence; letters are de-accented and HTML tags, copyright signs, punctuation, numbers, and non-alphanumeric characters strings are stripped; and all white space is removed from the strings. Regarding step 6, we match two articles within a given dataset if they share the same set of references, along with two other identifiers; at the moment we do not use references to match across datasets. If two documents have at least three identifiers in common, we treat these documents as identical. Once the first deduplication step (Figure 1) is complete, we use the processed data to the second deduplication step (Figure 2). We use the simhash fuzzy matching algorithm (Charikar et al., 2002; Manku et al., 2007) with a rolling window of three characters (*k* = 3) to match concatenated titles and abstracts of articles that share a publication year; documents that match according to this method are also considered identical.

**Figure 3:**
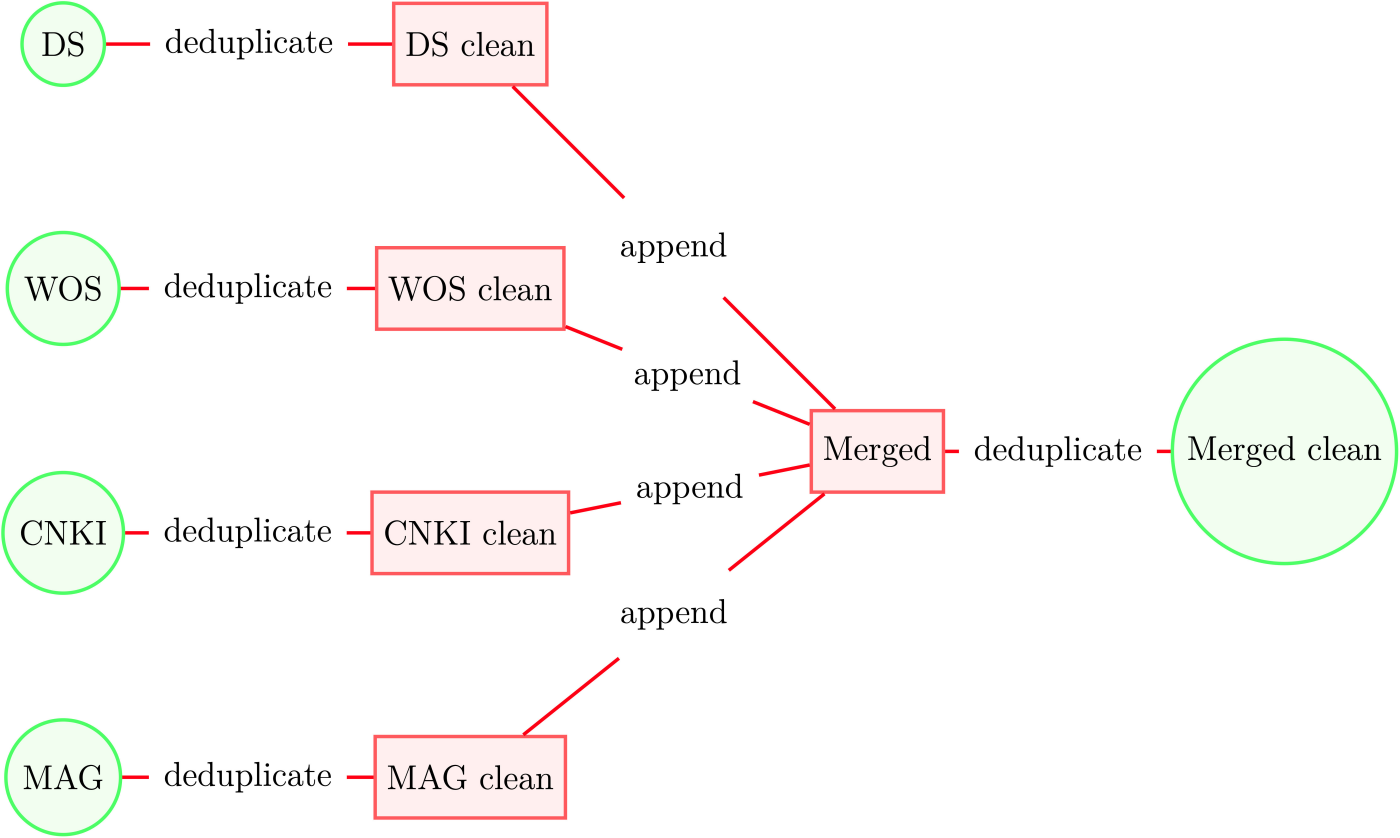
Construction of merged academic dataset.

We apply this deduplication process first to the original datasets and then to the merged dataset (see Figure 3). The deduplication process results in 237.6 million unique documents, of which 108.9 million were designated as articles by the original data sources and have at least one citation or reference, see Dunham et al. (2020) for additional details^3^. These 108.9 million articles constitute our academic dataset. From the academic data we select the 54.7 million core publications (as of October 2020) that have at least one citation and one reference as of June 2020—these data form the initial clustering model. After that, we assign a cluster ID to the remaining linked articles in the academic dataset using their references or citations to form a final set of clustered documents that contains 104.9 million articles.

The majority of the documents, including those indexed by CNKI, have English abstracts and titles, despite many being originally written and published in another language. Table 1 displays the total number of articles in the combined dataset that were written in various languages; the top seven most common languages are presented. The majority of global scientific articles have English language titles (67% of unique documents). The CNKI dataset, as acquired, requires the creation of a citation graph to map direct citations between articles^4^. To address this gap, a citation graph is created by matching references from the full-text of the CNKI articles to the article titles from CNKI, WOS, DS, and MAG. The simhash matching algorithm is again utilized. This approach matched about 50% of the non-Chinese citation strings from CNKI. For comparison, the WOS dataset has a match rate that is ~75%.

**Table 1:** Breakdown of document counts by languages (in millions), language labels are non-exclusive.

	**All languages**	**English**	**Chinese**	**German**	**French**	**Spanish**	**Portuguese**	**Japanese**
All documents	382.8	307.1	72.1	7.6	6.1	5.3	3.7	13.0
Unique documents	237.6	159.9	33.5	5.2	4.7	4.7	2.8	12.1
Citation graph	108.9	93.7	14.1	2.0	1.1	1.0	0.8	0.5
Core science	54.7	51.6	4.0	0.6	0.3	0.2	0.2	0.1
Clustered papers	104.9	92.0	11.4	1.9	1.1	0.9	0.8	0.5

*All Documents include all the scientific publications aggregated from Web of Science, Digital Science, Microsoft Academic Graph, and the Chinese National Knowledge Infrastructure; Unique Documents is the deduplicated set of scientific publications; Citation Graph contains the articles with at least one citation or one reference; Core Science contains the articles with at least one citation and one reference; and Clustered Papers contains the core science data clustered using the Leiden algorithm with additional articles assigned to clusters using direct-citation connections*.

The quality of reference disambiguation and the number of linked references tend to be lower among articles not written in English. For example, among 12.1 million documents with Japanese-language titles, only 500,000 articles have citations or references that we observe. For the articles included in the citation graph^5^, the share of articles that have an English language title rises to 86%. Among the core scientific articles that have at least one reference and at least one citation, the share of articles that have an English title peaks at 94%; this percent slightly reduces to 88% among the clustered articles we analyze. The number of clustered articles (displayed in the last row of Table 1) with titles in the top seven languages is 108.6 million, while the number of unique documents is 104.9 million. An article can present the abstract and title in multiple languages, though 92.1% of articles have only one language, 7.7% have two languages, and only 0.2% have more than two languages. Our analysis treats each article as one unit even if it presents information in multiple languages.

Table 2 breaks down the articles by the authors' country/region of affiliation for the four regions represented by our funding organizations of interest (China, the European Union, Japan, and the United States)^6^. If the authors of an article are affiliated with different countries, then the publication is included in both national accounts. Similarly, if the same author is affiliated with multiple countries, then the article receives a whole number count for all the countries noted in the affiliation. Among the 104.9 million clustered articles we analyze (see the last row of Table 2), 40.6 million articles lack country affiliation due to incomplete affiliation string parsing and disambiguation. The total national counts of China, the European Union, Japan, and the United States amounted to 54 million articles, while the remaining 10.3 million articles in the RCs were affiliated with countries or regions not mentioned above. For CNKI articles written in Chinese, we assume the authors are affiliated with China. In practice, this made a minimal difference, adding country affiliations for about 2% of the articles beyond those Chinese articles that had an explicit Chinese affiliation.

**Table 2:** Breakdown of document counts by country affiliation (in millions), country labels are non-exclusive.

	**All**	**Missing country**	**China**	**EU**	**Japan**	**United States**
All documents	382.8	193.7	31.4	60.3	10.9	56.7
Unique documents	237.5	157.6	12.6	24.8	4.5	23.9
Citation graph	108.9	44.0	10.9	20.6	3.7	19.3
Core science	54.7	10.0	6.4	15.2	2.7	14.2
Clustered papers	104.9	40.6	10.6	20.5	3.7	19.2

*All Documents include all the scientific article publications from Web of Science, Digital Science, Microsoft Academic Graph, and the Chinese National Knowledge Infrastructure, while Unique Documents implements article-level disambiguation on this set. Citation Graph contains the articles with at least one citation or one reference, Core Science contains the articles with at least one citation and one reference, and Clustered Papers contains the core science data clustered using the Leiden algorithm with additional articles assigned to clusters using direct-citation connections*.

We discard over half of the documents in the combined dataset prior to clustering because these documents do not have any observed citations and references, and thus, cannot be included in the citation graph. Documents with no country affiliations are most likely to be in this category. Out of 157.6 million such documents, only 44 million are included in the citation graph and only 10 million are included in the core science data subset. In Table 2, we see that most of the articles in the Japanese language are dropped. However, in Table 2 we see that the articles affiliated with Japan have much lower attrition rate than the articles with a title in the Japanese language. Out of 4.5 million documents affiliated with Japan, 3.7 million are included in the citation graph and in the set of clustered articles. Most of the articles written in the Japanese language are missing country affiliation (97%) and observed citations (96%), so in Table 2 they would be included in the column of articles with missing country affiliation. Most likely these documents are not scientific articles because they have no references, no citations, and no author affiliation information. This characteristic is most common among MAG and CNKI documents.

Table 3 presents the breakdown of documents by the six funding organizations of interest (EC, ERC, JSPS, NIH, NNSFC, and NSF). The majority of articles in our combined dataset do not acknowledge any funding organizations. While funding information may be incomplete for various reasons, we take the papers at face value and make no effort to infer the correct funding organization. We recognize that this missing data makes head-to-head comparisons between funding organizations impossible. Instead, we compare the areas of research that each funding organization supports relative to their median number of supported papers. We believe this is a more reliable comparative measure for analysis than using the total number of articles that acknowledge funding support.

**Table 3:** Breakdown of document counts by funding agencies (in millions), funding agency acknowledgments are non-exclusive.

	**All**	**No funder**	**EC**	**ERC**	**JSPS**	**NIH**	**NNSFC**	**NSF**	**Other funder**
All documents	382.8	328.1	2.3	0.7	2.1	8.0	11.3	2.3	32.3
Unique documents	237.5	219.3	0.7	0.2	0.7	2.9	4.2	0.7	10.7
Citation graph	108.9	90.9	0.7	0.2	0.7	2.9	4.0	0.7	10.5
Core science	54.7	39.7	0.6	0.2	0.6	2.5	2.6	0.6	8.7
Clustered papers	104.9	86.9	0.7	0.2	0.7	2.9	4.0	0.7	10.5

Of the 104.9 million clustered articles, 86.9 million did not acknowledge any funding, while 18 million articles did acknowledge at least one funding organization. Among the articles that acknowledge a funding organization, 9.2 million listed the funding organizations we analyzed (EC, ERC, JSPS, NIH, NNSFC, and NSF), and 10.5 million articles were funded by other funding organizations. The 18 million funded articles recognized 19.7 million funding events, indicating that some articles were funded by more than one funder, but this was relatively uncommon. Almost all of the articles that declared a funding organization are included in the citation graph and core science data subset. Notably, many NNSFC-funded articles did not have observed citations; however, 4.0 million are present in our cluster analysis due to their references, so the dataset is well-represented.

### 2.2. Inferring Research Clusters

From our scholarly literature dataset, we identify 55 million articles that have at least one citation and one reference to another article. We selected articles that were members of the solid scientific network (which includes references and citations). We picked a minimum in order to be the most inclusive of the papers where there is a clear signal of their network relationship. These documents that may not have any references (such as a book or newspaper article) can be added later if they are cited by the clustered papers. The documents that may not have any citations (such as an article that was just published yesterday) can also be added because of the references within the clustered papers.

Based on these direct citation links between these 55 million articles, we construct a network of 1.4 billion (10^9^) links. We cluster this direct citation network using the modularity-maximizing Leiden algorithm (Traag et al., 2019), a method whose accuracy has been characterized in Klavans and Boyack (2017b). Following the work of Klavans and Boyack (2017a), we infer a clustering solution targeting the average cluster size of several hundred articles. We run the Leiden clustering algorithm with 20 different starting resolution values ranging from 1 × 10^−20^ to 9 × 10^−2^. We compare each iteration's final network output and find a clustering resolution of 1.615 × 10^−4^ produces the average cluster size most similar to our target of several hundred articles. Overall, we identify 224,000 preliminary RCs with the average cluster size of 468 papers. The smallest RC contained two articles and the largest RC contained more than 20,000 articles.

Next, we erase RCs with fewer than 50 articles, as these clusters contain noisy data and were unstable throughout the clustering iterations, leaving 126,915 clusters in the network. We assign articles from these small clusters, articles that were never assigned a cluster (e.g., that had references or citations but not both), and outstanding articles that were indexed between June 2020 and October 2020 to these 126,915 RCs. We measure the number of in- and out-citation links between unclustered papers and each inferred cluster, and then assign 41 million remaining articles to an RC based on the maximal citation links. This returns 96 million articles with an assigned RC (a distinct cluster ID) and 13 million articles unassigned. With this updated network of RCs, we repeat the assignment process, resulting in the assignment of 9 million articles additional (of the 13 million unassigned articles) to multiple RCs. The remaining four million articles have weak citation connections; therefore, we stop the RC assignment process here to ensure an optimal clustering solution and do not include these in the final RC structure. Our final network of RCs contains 104.9 million articles from the four combined data sources.

We test the quality and stability of clustering solutions in two ways using modularity and review articles. Modularity is the difference between the edge density inside clusters to the expectation of such density with random cluster assignment, see more in Reichardt and Bornholdt (2006). Review articles present alternative topical definitions as the authors of review articles often cover a specific coherent topic. Klavans and Boyack (2017b) suggested using review article references as a basis of comparison. We identify review articles as all articles with the number of references between 100 and 1,000 references. We measure the share of references from the 1.6 million review articles (1.5% of all articles) that appear in the same cluster as the review article. We estimated 10 randomly seeded clustering models for the periods 2005–2014, 2005–2015, and 2005–2016 and calculated the two quality measures for each model. The clustering solutions show great stability, the standard deviation in modularity is 0.0018 (mean modularity is 0.183), while the standard deviation of the average share of review references belonging to the review articles is 0.0025 (mean share is 0.243).

**Figure 4:**
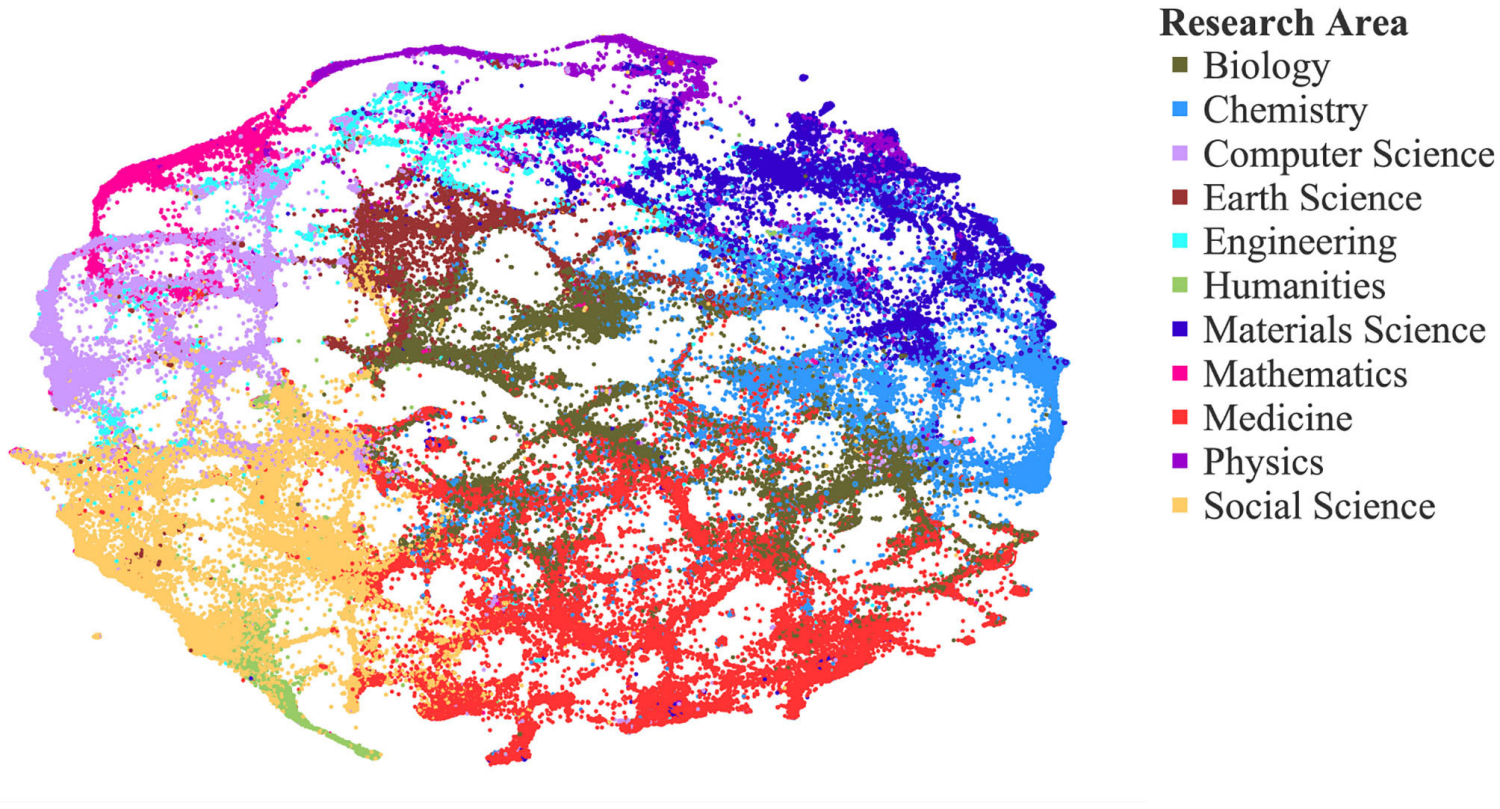
Map of science: 126,915 active research clusters (RCs) creating an inferred structural basis for analysis from direct citation links between tens of millions of articles across four data sources.

We display our RC network in Figure 4, with each RC categorized by its corresponding research area. The map presents 122,110 clusters that have at least five publications in the last 5 years. To create the map, we calculate cluster-cluster relatedness. For a given cluster, we calculate the number of direct citation (DC) connections between the cluster and the other RCs. To reduce noise and improve computational speed, we keep the top 20 strongest connections for each cluster. We measure the strength of connections between two RCs, *A* and *B*, as: *Strength*_*A*&*B*_ = *DC*_*A*&*B*_/*DC*_*A*_, where *DC*_*A*&*B*_ is the total number of undirected edges between A and B and *DC*_*A*_ is the number of external edges for A.

We calculate a map layout using Distributed Recursive Graph Layout (Martin et al., 2011) in the iGraph Python package, which positions clusters that are most connected close to each other. The map coloring is based on the aggregation of 19 level-0 (most aggregated) scientific fields from Microsoft Academic Graph into 11 even more aggregated fields. The scientific field of a cluster is the most common scientific field among its papers. If no paper in a cluster is classified by MAG, we assign a scientific field based on the most frequent appearance in the top 100 most connected clusters. The cluster connectedness is the share of direct citation connections between the two clusters as described above.

### 2.3. Forecasting Extreme Growth

We use the methods from Klavans et al. (2020) to predict extreme growth clusters for our inferred RCs. Following Klavans et al., we estimate several backcasting models in order to predict the probability that, for a given RC, its share of the total scientific production will grow by at least 8% annually over a 3-year period. Only clusters with at least 20 publications in the last 12 months are stable and active enough to predict their growth; therefore, we restrict our attention to forecasting the future state for the 55,975 clusters that satisfy this criterion. On average 3–4% of clusters were forecasted to reach the level of extreme growth.

Four factors are used to predict the extreme growth of each cluster:

- **Paper Vitality**: Defined as pvit $=\frac{1}{N_{c}}\sum \frac{1}{age_{paper}+1}$, where *N*_*c*_ is the number of papers in a cluster, Paper Vitality measures how “young” the articles are in a given cluster.
- **Citation Vitality**: Defined as cvit $={\left(\frac{1}{N_{c}}\sum \frac{1}{N_{r}}\sum \frac{1}{1+age_{reference}}\right)}^{1/4}$, where *N*_*c*_ is the number of papers in a cluster and *N*_*r*_ is the number of times the paper was referenced (i.e., the age of reference is the difference between the current year at calculation, 2020, and the year the article was published that references the article in the cluster).
- **Top 250 sources**: The number of publications in the top 250 journals and conferences based on Elsevier's CiteScore metric. For each journal we take the number of publications in 2016–2018 and measure the number of citations these publications receive in 2019. Then we divide the number of publications by the number of citations to get the score. We rank the journals/conferences according to this score and get the list of top 250 sources. For each cluster we calculate the logarithm of the number of publications in the top 250 sources: Top250J = *log*(*N*).
- **Growth Stage**: For each cluster, we determine the year where the cluster's share of the global scientific output reached its maximum. Then we calculate the number of years that have passed since this peak year until current year at calculation, 2020 [i.e., growth stage = 1/(1+2020 − *year*_*peak*_)].

All variables are standardized by subtracting the mean and dividing by the standard deviation. Then we predict extreme growth probability based on the regression coefficients estimated by Klavans et al. (2020):

(1) $$\begin{array}{r} \text{Extreme Growth Probability}=0.473*\text{pvit}+0.113*\text{Top250J}\\ +\;0.292*\text{GrowthStage}+0.1*\text{cvit}. \end{array}$$

We then rank all clusters based on the probability of high growth and select the top 2,000 (3.6%) clusters. This threshold maps to an expected yearly share increase of publications at or above 8% per year.

RCs that do not meet the 8% threshold of extreme growth encompass RCs that have minimal growth, no growth, and reduction in growth. We refer to these RCs as having “typical growth.” The forecasting model is estimated from the data observed in late October 2020. We assume a 150-day lag between publication and the appearance of an article in our database. Thus, the forecasting model predicts which clusters are expected to experience extreme growth between May 2020 and May 2023, as it would be observed in late October 2023.

In Klavans et al. (2020), the authors make a 3-year extreme growth forecast using the relatively clean English Scopus metadata and citation data from Elsevier. They measure a Critical Success Score (CSI) score (among other measures) to characterize the forecasting accuracy of rare events: *CSI* *score* = (*truepositives*)/(*truepositives* + *falsepositives* + *falsenegatives*). The CSI scores for Klavans et al. (2020) vary between 0.2 and 0.33. Our forecasting task is more difficult because the linkages between the four diverse datasets are more sparse, which results in a less connected citation graph. In sparse citation graphs, the clusters are less pronounced and their growth is harder to predict.

To characterize CSI in our model, we create three backcasting datasets, where we take three historical snapshots and then evaluate the accuracy of the forecasting model using known future observations: 2014–2017, 2015–2018, and 2016–2019. For each time period, we generate nine randomly seeded clustering maps to test both the accuracy and stability of backcasting results. The average CSI results for all clusters is 0.17, and the results from different clustering maps are tightly bounded between 0.16 and 0.19. The average CSI for AI RCs is 0.19, but it varies between 0.08 and 0.43. The main source of variation is the changing composition of AI clusters due to the stochastic nature of the clustering algorithm. We define clusters to be AI if more than 50% of the articles in the cluster are classified as AI/ML (see the classification discussion below).

### 2.4. Funding Organization Disambiguation

In order to analyze a funding organization's funding portfolio, we need to identify the articles that acknowledge any of the six funding organizations we selected. These organizations have many variations in how they are acknowledged (e.g., organization aliases and misspellings). To ensure the most representative lists of articles for each funding organization are included, we implement two methods to disambiguate organization names: (1) the Global Identifier Research Database^7^ (GRID) identifiers that are automatically linked in the Dimensions and MAG datasets and (2) regular expression matching to expand coverage in the GRID-friendly datasets and to include acknowledgments in the CNKI and Web of Science datasets.

The first disambiguation method uses GRID identifiers associated with the funding organization in order to include any departments or sub-organizations within an organization; for example, NSF has seven directorates that are often listed as the funding organization. We identify the GRID identifiers for the six funding organizations of interest and use GRID to identify their sub-organizations.

The second disambiguation method uses regular expression string matching in the funding organization field of our dataset to catch any mentions of an organization that are variations on its official name format. We searched for strings that contained an organization's name (e.g., Japan Society for the Promotion of Science), as well as the organization's acronym (e.g., JSPS). We included misspellings for “European Commission” and restricted the National Science Foundation searches to include “US” or “U.S.,” since many countries have a National Science Foundation.

### 2.5. AI/ML Article Classification

We classified articles as AI/ML according to the predictions from the SciBERT machine learning classifier (Devlin et al., 2018) performed by Dunham et al. (2020). The classifier was trained on article abstracts and titles from arXiv^8^ where the authors labeled their articles with at least one of the following fields: cs.AI, cs.LG, stat.ML, and cs.MA. While cs.AI is strictly AI, cs.LG, stat.ML, and cs.MA are heavily AI/ML-related. Articles labeled with other fields are considered non-AI/ML examples. In addition, Dunham et al. (2020) use the SciBERT model to classify three sub-fields of AI: Computer Vision (CV), Natural Language Processing (NLP), and Robotics (RO). Each of the sub-fields was predicted using an independent classifier, allowing for rare cases where an article can be classified as a sub-field of AI (e.g., RO) but not as AI/ML-related. Articles can also be classified as general AI/ML and not as any of the three sub-fields.

Once the model is trained on the arXiv dataset, Dunham et al. (2020) use it to classify the rest of the articles from the Dimensions, WOS, and MAG data sources. Articles from 2010 to 2020, with abstracts and titles available in English (nearly 88%), are classified using this method. Since the AI/ML classification model was designed for English articles, there was an additional general AI classification method for the Chinese articles (10.9% of all articles) that uses a search query for relevant terms and phrases. If an article does not contain any of these Chinese terms and phrases, it is classified as non-AI.

An RC was classified as AI if more than 50% of its articles were classified by the SciBERT model or by the Chinese-language AI query. The Chinese-language articles are classified as AI/ML-related when the following regular expression query returns TRUE^9^. English language articles published prior to 2010 are assumed to be non-AI. Out of the 126,915 RCs, there are 600 of these RCs where the share of AI/ML-classified papers are above 50%, and this subset of RCs enables our analysis of AI scientific research funding.

## 3. Results

For our analysis, we are interested in RCs with a strong focus in AI/ML. If at least 50% of articles in an RC are classified as AI/ML, we label the cluster as AI. Our completed network of RCs contains a total of 126,915 clusters, of which 600 are labeled as AI clusters and had at least 20 publications between May 2019 and May 2020. Using the 8% annual forecasted growth threshold to classify an extreme growth cluster, we forecast that 161 AI-relevant RCs will experience extreme growth, which contain 344,022 out of 1,032,850 (33.4%) publications in these 600 clusters. In Table 4, we count the number of publications in these clusters by region and by funder to understand which funding organizations and regions target AI/ML research areas and extreme forecasted-growth research areas.

**Table 4:** Publications in AI research clusters: production by region, funding organization, and presence in extreme growth RCs.

**Region**	**Total articles**	**Articles in extreme growth RCs**	**Share of articles in extreme growth RCs (%)**	**Funder Org**.	**Funded articles**	**Median share funded (%)**	**Articles funded in extreme growth RCs**
CN	355,068	127,378	35.8	NNSFC	256,526	19.3	102,326
EU	187,624	53,867	28.7	EC	18,731	1.3	4,702
				ERC	7,037	0.2	2,999
JP	40,430	11,132	27.5	JSPS	16,139	1.0	4,954
U.S.	183,355	72,259	39.4	NIH	18,821	0.4	8,223
				NSF	22,984	1.7	9,327

*Article content is aggregated from Web of Science, Digital Science, Microsoft Academic Graph, and the Chinese National Knowledge Infrastructure databases and includes publications from 600 research clusters with at least a 50% share of AI-relevant articles and contain at least 20 articles published between May 2019 and May 2020*.

We analyze each funding organization by their contribution to AI research clusters and identify those that are forecast to experience extreme growth. For each funder we calculate the shares of papers funded in each of the 600 AI research clusters considered. Due to variance in funding organization reporting in research publications, we calculated a median share of article funded by each funder and used it as our measure of focus. We measure a funding organization's level of contribution as follows: (1) Identify the share of articles in AI clusters on which the funding organization is acknowledged (see the “Median Share Funded” column in Table 4); (2) Set the median share of AI/ML-classified articles funded as the threshold for the funding organization to be labeled a contributor to a given research cluster—each funder is thus a contributor to the half of the clusters in which they have greater than their median share; and (3) Define the level of contribution as the fraction of clusters in which the funding organization is acknowledged.

Table 4 shows that NNSFC funds 256,526 articles of the 355,068 articles published by Chinese authors. Furthermore, NNSFC has the highest median share of AI clusters funded at 19.3%; NSF, with the second-highest median share of AI clusters funded, is significantly lower at 1.7%. Overall, NNFSC funds 75% of all the funded articles in this subset of RCs. The 161 extreme growth AI clusters contain 33.3% of the articles in all AI clusters considered. We find that the United States and China have higher-than-average shares of AI/ML-classified articles in extreme growth clusters at 39.4 and 35.8%, respectively. Alternatively, the EU and Japan have below-average shares of AI/ML-classified articles in extreme growth clusters at 28.7 and 27.5%, respectively.

We find that the NSF and ERC are the top two funding organizations for extreme growth AI RCs, with 35 and 36.2% funding contributions, respectively (see Table 5). While the NSF and ERC are frequent contributors to extreme growth clusters, their national funding contributions are smaller, with only 12.0 and 3.8% funding contributions to AI/ML-classified papers published in the United States and the EU, respectively. Alternatively, NNSFC has a slightly lower contribution to extreme growth AI RCs (30.7%) but is acknowledged by 69% of AI/ML-classified research papers published by authors representing Chinese organizations. Additionally, we observe a significant difference in funding contributions between the ERC and EC in Table 5. The ERC targets extreme growth AI RCs at a higher level than the EC does, with a 14.3% difference in share of funding. The overall share of extreme growth RCs is 26.8%, indicating that all funders, except for the EC and Japan, support extreme growth RCs at a rate slightly above random chance.

**Table 5:** Funding organization participation in extreme growth AI research clusters (RCs).

**Region**	**Funder**	**Share of extreme growth RCs (%)**	**Share of typical RCs (%)**	**Level of contribution *Region* (%)**
CN	NNSFC	30.7	69.3	70.2
EU	EC	21.9	78.1	9.3
	ERC	36.2	63.8	3.8
JP	JSPS	26.7	73.3	36.7
U.S.	NIH	30.0	70.0	8.1
	NSF	35.0	65.0	12.0

*Article content is aggregated from Web of Science, Digital Science, Microsoft Academic Graph, and the Chinese National Knowledge Infrastructure databases and includes publications from 600 research clusters with at least a 50% share of AI-relevant articles and contain at least 20 articles published between May 2019 and May 2020*.

Recognizing that funding organizations have varying motivations and goals, we further investigate the differences in their funding portfolios. Table 6 presents the results for the top five AI RCs, as well as their sub-fields: Computer Vision (CV), Natural Language Processing (NLP), and Robotics (RO). We use the classification labels from section 3.5 and compute the percent of CV, NLP, and RO articles for each cluster. We label a cluster as CV, NLP, or RO if the percent of a given AI sub-field was at least 25% and greater than all other sub-fields. For example, if %_*NLP*_ ≥ 0.25, %_*NLP*_ ≥ %_*CV*_, and %_*NLP*_ ≥ %_*RO*_, then the cluster is labeled as NLP. If a cluster has <25% of articles in any of the three AI sub-fields, we label the cluster as AI/ML to represent general AI/ML research not covered by the other three areas.

**Table 6:** Top five extreme growth research clusters for each category of AI.

**AI category**	**RC rank and description**	**Funders contributing above their median share**	**CN share (%)**	**EU share (%)**	**JP share (%)**	**U.S. share (%)**
AI/ML	1. Interpretable machine learning	NSF, NIH, EC, ERC, JSPS	9	32	4	32
	2. Multiple attribute decision	NNSFC	56	5	0	3
	3. Overparameterized NNs	NSF, ERC	9	13	2	55
	4. Bias/Fairness in AI/ML	NSF, ERC, EC, JSPS, NIH	1	28	2	48
	5. Actor-critic models	JSPS, ERC	22	20	5	30
CV	1. Deep learning (GANs)	NNSFC, NSF, JSPS, NIH, ERC	30	17	5	25
	2. R-CNN object detection	NNSFC	58	9	3	11
	3.3D object classification	NNSFC, NSF, ERC, EC, JSPS, NIH	34	18	3	22
	4. Adversarial neural networks	NSF, NIH, ERC	24	15	3	36
	5. Depth estimation	NNSFC, NSF, EC, ERC	35	18	3	20
NLP	1. Natural language inference	ERC	23	13	3	35
	2. Text completion	NNSFC, ERC, NIH	28	20	8	22
	3. Neural conversation models	JSPS	35	12	6	26
	4. Cross-lingual word embeddings	JSPS, ERC	21	25	5	27
	5. Hate speech detection	NSF, EC, JSPS	4	28	2	27
RO	1. Soft robotics	NNSFC, NSF, JSPS, EC, NIH, ERC	24	19	10	23
	2. Imitation learning	NSF, EC, NIH, JSPS, ERC	9	22	4	44
	3. Gaussian processes	ERC, EC, JSPS, NSF	5	29	4	20
	4. Visual odeometry	NNSFC, NSF, EC, ERC, NIH	33	18	1	26
	5. Autonomous driving	NNSFC, NSF	24	19	3	27

*All funders are listed in order from highest contributor to lowest*.

**Figure 5:**
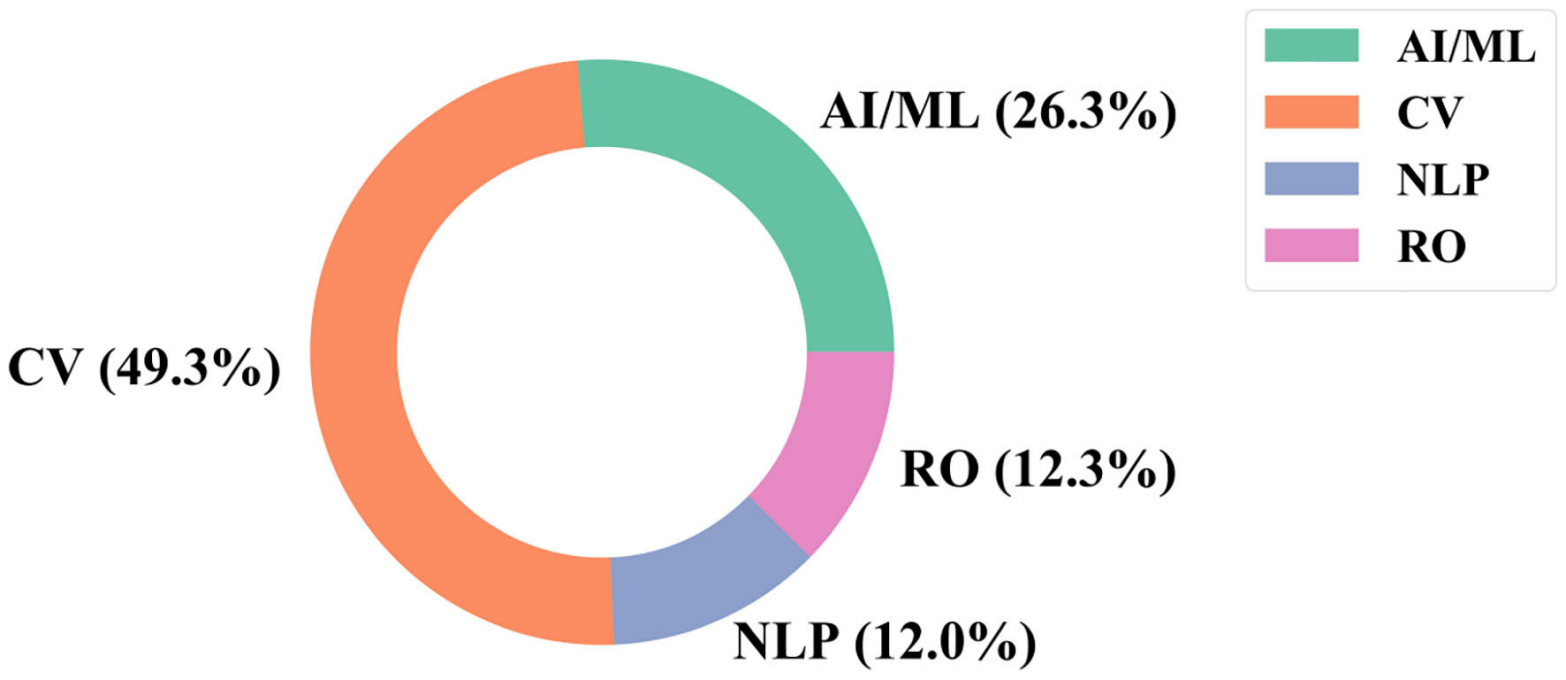
Breakdown of research clusters (RCs) by AI field.

**Figure 6:**
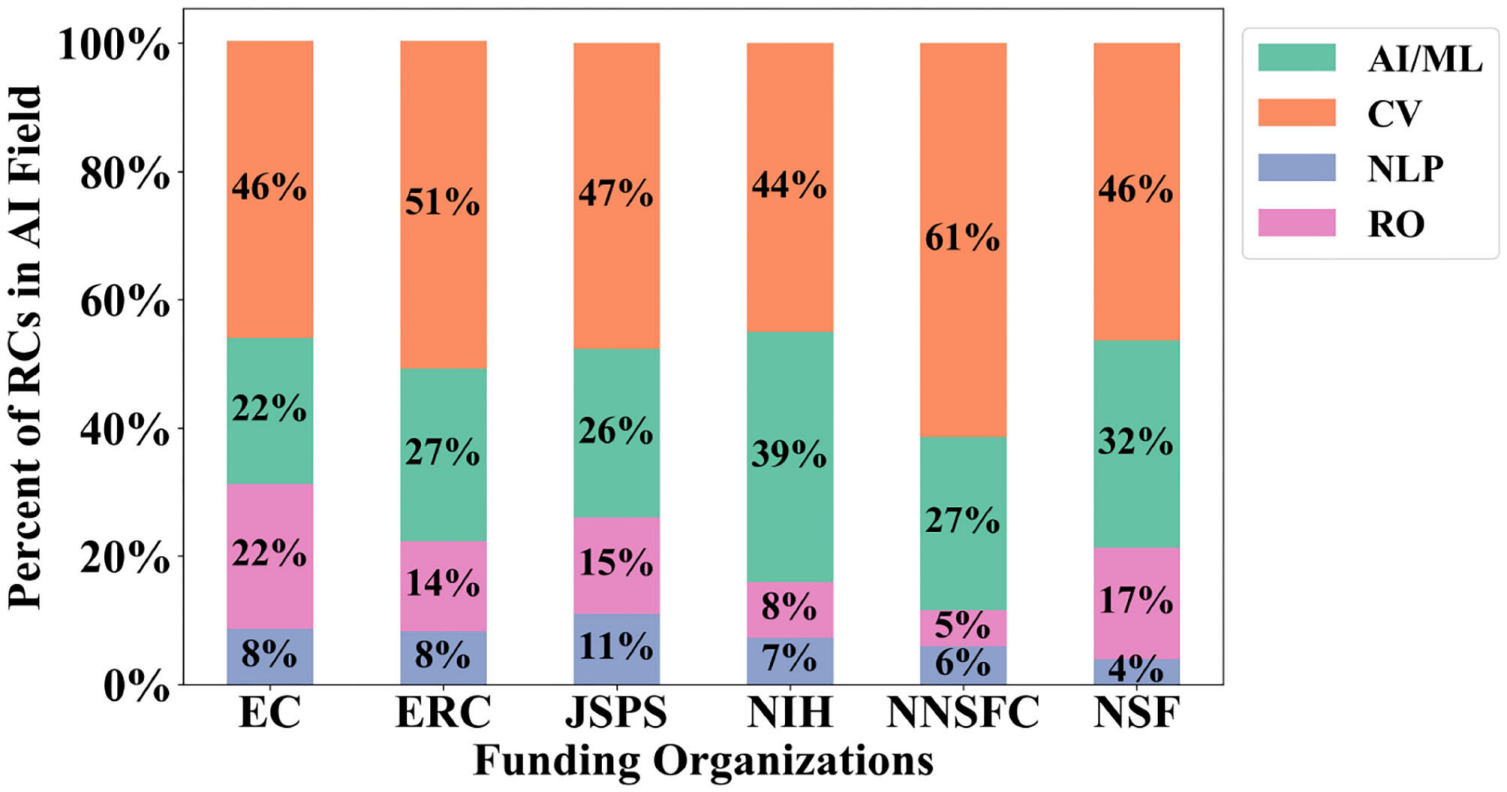
Research cluster (RC) funding portfolio breakdown by funder and AI field.

Figure 5 shows the percent of the 600 large AI RCs that are classified into each sub-field of AI, including the general AI/ML category^10^. CV has a significant lead over the other fields, with 49.3% of the extreme growth AI RCs classified under its research area, and NLP and RO are almost evenly represented in the extreme growth clusters with 12% of the RCs classified in each respective research area. Figure 6 provides the results for each funding organization's support across the AI RCs by the AI fields we assigned. In general, each funding organization contributes above their median share to CV the most, with general AI/ML, RO, and NLP following, respectively. The exception is the NNSFC, who contributes above their median share to NLP (18 RCs) slightly more than RO (17 RCs).

Table 6 provides more insight into which sub-fields of AI each funding organization supports, as well as specific research areas within those sub-fields. We also include the share of articles published by authors in the regions of the funding organizations we analyze. The CV sub-field provides a noticeable example of this, as NNSFC is the leading funder for all but one CV RC, with NSF as the second highest contributor. It is also of note that most funders are contributing above their median share to all of the top five extreme growth RO RCs, whereas NLP has the smallest number of supported papers. CV RCs are the second most supported by all six funding organizations, with general AI/ML and NLP following accordingly. NNSFC only funds one cluster in each of the top five extreme growth RCs for general AI/ML and NLP. Additionally, all funding organizations except for NNSFC contribute above their median share to the Bias/Fairness RC in general AI/ML.

While NNSFC is the leading funding organization for 45% of the top extreme growth RCs, there are several areas that other funding organizations lead that are of note. JSPS leads in two out of the five NLP extreme growth RCs, and is the only funder contributing above its median share in the third RC (Neural conversation models). The ERC contributes to three out of the five extreme growth NLP RCs as the second highest contributor, except for the first cluster (Natural language inference) where they are the only funder. The leading funding organization for three out of the five for AI/ML extreme growth RCs is NSF, with the ERC as the second highest contributing funder for three out of the five top RCs.

## 4. Discussion

### 4.1. Analytic Conclusions

We are particularly interested in the growth of clusters since it represents the growing importance of a particular research area. Organizations that finance scientific research are typically interested in knowing if their funds support growing or stagnating areas of science. Here we provide insight into the funding portfolios of six organizations with respect to extreme growth AI RCs.

We find a significant difference in the amount of support the national funders provide to AI and their focus on extreme growth clusters. NNSFC supports most of the AI research in China and the largest fraction of global AI research. The NSF and ERC are similar in that they have a smaller share of AI research, but have a high focus on extreme growth clusters. The EC, in contrast, has the least focus on extreme growth. Whether these differences are due to organizational policies that favor risk-taking is an open question. While all of these research organizations claim that they fund higher-risk research, the initial analysis above suggests that the observed rate is only slightly above random chance.

Analyzing specific sub-fields of AI (general AI/ML, CV, NLP, and RO) in the RCs that are forecast to experience extreme growth, we find that the NSF and ERC are active (i.e., contributing above their median share) to general AI/ML RCs. We also highlight the Bias/Fairness RC in the set of general AI/ML RCs, since all funding organizations except NNSFC contribute above their median shares. Notably, China only published 1% to this RC, despite publishing 48.6% of the articles in the extreme growth RCs.

The research areas of CV and RO are actively supported by all funding organizations. CV publications are dominated by Chinese authors, while the RO publications are more widely distributed across the geographic areas we consider, with the United States often publishing the most articles. The research area of NLP is actively supported by Japan and the EU, though Chinese and American authors tend to publish the most papers in this area.

These organizations have many factors to consider when awarding funding to a research area, and funding an extreme growth area of research may not always be the top priority. For example, the use of robots in elder care is a high priority for Japan (Tanioka, 2019), resulting in the JSPS prioritizing RCs that focus on the development of robotic technology regardless of the forecasted growth of the RCs. Knowing these priorities would allow for more accurate comparisons among funding organizations and is a promising area of research for future work.

### 4.2. Implications

Improving the portfolio-level management of the public-sector funding organizations (Smit and Trigeorgis, 2006; Wallace and Ràfols, 2015; Vonortas and Ràfols, 2018) may be worth considering as the dominance of government-sponsored research and development (R&D) investment is challenged by an increasing array of actors and an increasing pressure for documenting research outcomes (Flagg and Harris, 2020). Expanding from processes that consider primarily the merit of each project individually to one that also weighs an appropriate mix of portfolio-level factors could positively impact each funding organization's ability to accomplish its mission. The optimal method to balance technical merit, social value, and comparative advantage within the context of the investment landscape needs to be tailored to each organization. However, the new methods introduced by this paper provide key contextual insight that may provide valuable insight to all funding organizations.

It is important to understand the interaction between a given funding organization's level and type of support in a set of RCs. The results of our RC model and linked funding data offer promising directions for future work by exploring questions that have historically been very difficult to answer. Examples include:

- Does a funding organization provide seed funding or otherwise drive the research cluster activity or ride an existing wave of activity (e.g., the percentage of papers that acknowledge funding support at various stages in its development)?
- Is a funding organization providing the lion's share of regional support for research (e.g., NNSFC) or is it part of a plurality of regional AI research funders (e.g., NIH)?
- If funding in a given area were not provided, would the desired goals of the project(s) in question still be achieved on a reasonable timeline based on RC activity and extreme growth forecasts?
- What is the right mix of typical vs. extreme growth research areas of emphasis for a given funding organization? How does the extreme growth forecast relate to project risk (e.g., lower risk if part of a set of extreme growth research clusters or higher risk because the methods are newer and may not work)?

We do not answer these questions here, but leave them to future work.

Portfolio management is often driven by the need to address prioritized societal needs, such as health, security, environment, economic, and social well-being; however, insights to questions like those asked above do not require top-down funding decision management or a quantitative model of return on investment. Instead, the research program directors can proactively inform decisions to expand or redirect support based on the dynamics of the research landscape in the context of funding organizations. These dynamics are now clearly laid out and the related forecasts can be calibrated to best inform the decisions.

For example, a decision to invest in the development of autonomous control of waterborne vehicles using reinforcement learning techniques can be quickly informed by the level of current activity in this problem area, the 3-year future growth rate of the relevant research clusters, an analysis of the current relative worldwide funding support by region, an institutional diversity analysis, and a quick review of the degree to which the desired program goals are already being pursued even if the program is never started by the funding organization. The RC structure and extreme growth predictions provide unique insight that can help decision-makers determine the right mix of breakthrough research, fundamental research, or translational research. The portfolio can then be further balanced by the proper risk vs. reward considerations.

### 4.3. Limitations

While the scale and scope of the methods described above represent a step forward, there are many areas that require additional work and testing before they can be officially deployed by a funding organization to inform their portfolio. First, the RC structure creates a useful frame of reference when it is constructed; however, it is unclear how long this RC structure will remain the best way to represent the scientific landscape. If a unique line of inquiry around some problem very quickly grows within 1–3 years, then it is possible the existing RC structure that is recomputed from scratch every 3–5 years may not adequately reflect this area of innovation. Secondly, what a human considers a reasonable aggregation of research (e.g., autonomous ground-based vehicle control) may be split across multiple RCs in this representation; therefore, care must be taken when interpreting a few extreme growth RCs in this broader area without considering others that are not growing as quickly. Third, extreme growth forecast accuracy needs further exploration and calibration over multiple years to gauge its level of reliability for each agency decision. Lastly, the analysis needs to be customized for the needs of each funding organization. These limitations are currently topics of continuing research.

## Data Availability Statement

The data analyzed in this study is subject to the following licenses/restrictions: the majority of datasets used by this paper are commercially licensed and thus cannot be shared publicly. The MAG data set can be obtained through access to the Azure cloud, but, again, the authors are not authorized to distribute these data. Requests to access these datasets should be directed to WOS: a.patel@clarivate.com; Dimensions: Duane@uberresearch.com; CNKI: Tammy.Byrne@eastview.com; MAG: https://docs.microsoft.com/en-us/academic-services/graph/get-started-setup-provisioning.

## Author Contributions

IR implemented the methods and co-wrote the paper. AT performed the results analysis, created the figures and tables, and co-wrote the paper. KB and RK played a fundamental role in developing and testing the methods and provided key input into the paper. DM wrote the abstract and discussion of the paper, edited the paper, oversaw the work, and acquired the resources for the research. All authors contributed to the article and approved the submitted version.

## Conflict of Interest

RK and KB were employed by the company SciTech Strategies, Inc., and assisted this project on a paid contract from CSET. The remaining authors declare that the research was conducted in the absence of any commercial or financial relationships that could be construed as a potential conflict of interest.

## Footnotes

^1^The WOS indices utilized by this study include the following: Science Citation Index Expanded (January 2000–October 2020), Social Sciences Citation Index (January 2000–October 2020), Arts and Humanities Citation Index (January 2000–October 2020), Conference Proceedings Citation Index-Science and Technical (January 2000–October 2020), Conference Proceedings Citation Index-Social Sciences and Humanities (January 2000–October 2020), Book Citation Index-Science (January 2005–October 2020), Book Citation Index-Social Sciences and Humanities (January 2005–October 2020), Emerging Sources Citation Index (January 2015–October 2020), Chinese Science Citation Database (January 2000–October 2020), and Russian Science Citation Database (January 2005–October 2020).

^2^The full combined corpus includes patents and dataset descriptions that are not counted as part of these 382.8 million documents.

^3^The Python code to match articles is available at https://github.com/georgetown-cset/public-article-linking.

^4^All CNKI content is furnished by East View Information Services, Minneapolis, MN, USA.

^5^To be included in the citation graph, an article needs to have at least one reference or citation.

^6^The European Union includes the United Kingdom for the purpose of this study.

^7^https://www.grid.ac/

^8^https://arxiv.org/

^9^https://github.com/georgetown-cset/frontiers_funders_2020/blob/main/regular_expression_AI_query.txt

^10^Sub-field classification is not available for Chinese-language AI/ML-classified articles.
